# Thalamic Modulation of Cingulate Seizure Activity Via the Regulation of Gap Junctions in Mice Thalamocingulate Slice

**DOI:** 10.1371/journal.pone.0062952

**Published:** 2013-05-14

**Authors:** Wei-Pang Chang, José Jiun-Shian Wu, Bai-Chuang Shyu

**Affiliations:** 1 Graduate Institute of Life Sciences, National Defense Medical Center, Taipei, Taiwan, Republic of China; 2 Institute of Zoology, National Taiwan University, Taipei, Taiwan, Republic of China; 3 Institute of Biomedical Science, Academia Sinica, Taipei, Taiwan, Republic of China; St. Joseph's Hospital and Medical Center, United States of America

## Abstract

The thalamus is an important target for deep brain stimulation in the treatment of seizures. However, whether the modulatory effect of thalamic inputs on cortical seizures occurs through the modulation of gap junctions has not been previously studied. Therefore, we tested the effects of different gap junction blockers and couplers in a drug-resistant seizure model and studied the role of gap junctions in the thalamic modulation on cortical seizures. Multielectrode array and calcium imaging were used to record the cortical seizures induced by 4-aminopyridine (250 µM) and bicuculline (5–50 µM) in a novel thalamocingulate slice preparation. Seizure-like activity was significantly attenuated by the pan-gap junction blockers carbenoxolone and octanol and specific neuronal gap junction blocker mefloquine. The gap junction coupler trimethylamine significantly enhanced seizure-like activity. Gap junction blockers did not influence the initial phase of seizure-like activity, but they significantly decreased the amplitude and duration of the maintenance phase. The development of seizures is regulated by extracellular potassium concentration. Carbenoxolone partially restored the amplitude and duration after removing the thalamic inputs. A two-dimensional current source density analysis showed that the sink and source signals shifted to deeper layers after removing the thalamic inputs during the clonic phase. These results indicate that the regulatory mechanism of deep brain stimulation in the thalamus occurs partially though gap junctions.

## Introduction

Seizures affect 1% of the population worldwide, and 30% of affected patients have drug-resistant epilepsy [1]. Although synchronization is the hallmark of epilepsy, this property of neuronal networks has largely been ignored as a therapeutic target [2]. Evidence shows that gap junctions are involved in pathophysiological hyper-synchrony during seizure activity [3]–[6]. Therefore, growing studies seek to regulate gap junctions to modulate seizures. Gap junctions exist between interneurons in the neocortex [7] and are important in regulating synchrony between interneurons. Gap junctions are also expressed on glial cells [8]. The role of glial cells in seizures is to regulate the ionic concentration of the extracellular space and prevent the accumulation of potassium-generating neurons that become more excitable [9], [10].

Frontal lobe epilepsy (FLE) is the second most prevalent type of seizure [11]. Epilepsy in the anterior cingulate cortex (ACC) is a part of epileptic syndromes of frontal lobe origin and are usually classified as simple partial [12]. Cingulate seizures are associated with changes in autonomic function, motivation, and thought [13]. Epilepsy that occurs in this brain area may be attributable to enhanced γ-aminobutyric acid (GABA) function [14]–[16]. The possible GABAergic inhibitory mechanisms that contribute to epileptogenesis include the resetting of synchronization [17], direct excitatory effects of axo-axonic interneurons on layer II/III pyramidal cells [18], and changes in the reversal of GABA potential [19]. Although the pharmacological mechanisms of FLE have been characterized *in vitro* [20], the role of gap junctions in FLE, especially in the modulation of the spatiotemporal properties of seizure-like activity and changes in frequency distribution, have not yet been studied.

The thalamus supports the propagation and synchronization of limbic seizures [21]–[23] and plays a pivotal role in the remote control of seizure activity. Therefore, growing clinical studies have selected the thalamus as a target for deep brain stimulation (DBS) [24]. Electrical synapses in the thalamocortical system are strong. When electrically coupled cells in the neocortex are excited by thalamic inputs, they typically display strong synchrony of both subthreshold voltage fluctuations and spikes [25]. The ACC is heavily connected with the medial thalamus (MT) [26], [27]. Our recent study showed that inputs from the MT modulated seizure-like activity in the ACC [28]. Thus, we hypothesized that the modulatory effect of MT inputs on cingulate seizures occurs through the regulation of gap junctions. The present study used a novel thalamocingulate slice preparation [29]–[31] to address this issue. The drug-resistant seizure-like activity was induced by the application of 4-aminopyridine (4-AP; 250 µM) and a low dose of bicuculline (5 µM) [32]. Different types of gap junction blockers were applied before and after the thalamic inputs were removed to test the interaction between the MD and gap junctions in the ACC in modulating seizure-like activity.

## Materials and Methods

### Animals

Eight-week-old male C57BL/6J mice were used. The experiments were approved by the Academia Sinica Institutional Animal Care and Utilization Committee (IACUC, Permit Number: RMiRaIBMSB2011086) and all of the research procedures conformed to the guidelines of the National Institutes of Health. We made efforts to minimize animal suffering and the number of animals used.

### Slice preparation

The animals were anesthetized with 4% halothane in pure oxygen. The brains were quickly removed and cooled in chilled, oxygenated artificial cerebrospinal fluid (aCSF; 124 mM NaCl, 4.4 mM KCl, 1 mM NaH_2_PO_3_, 2 mM MgSO_4_, 2 mM CaCl_2_, 25 mM NaHCO_3_, and 10 mM glucose, bubbled with 95% O_2_ and 5% CO_2_). Slice sections that contained the pathway from MT to ACC were prepared according to a previously method established (Lee *et al.*, 2007). The slices were then incubated in oxygenated aCSF at room temperature for 1 h. A single slice was then transferred to the recording chamber and kept at 32°C under continuous perfusion (12 ml/min) of oxygenated aCSF.

### Multielectrode array recording

Multielectrode arrays (MEAs) were used to record the spatiotemporal properties of epileptiform activity. Two types of MEA were used: 6×10 planar MEA (electrode diameter, 30 µm; electrode spacing, 500 µm; impedance, 50 kΩ at 200 Hz; Multichannel System, Reutlingen, Germany) and 8×8 MEA (pyramidal-shaped electrode diameter, 40 µm; tip height, 50 µm; electrode spacing, 200 µm; impedance, 1000 kΩ at 200 Hz; Ayanda Biosystems, Lausanne, Switzerland). The data were acquired using MC Rack software (Multi Channel Systems) at a 10 kHz sampling rate.

### Paired extra- and intracellular recording

Borosilicate glass pipettes for extracellular recording (5–10 MΩ) and intracellular pipettes (120–200 MΩ) were pulled using a P-97 puller (SUTTER Instrument, Novato, CA, USA). Extracellular pipettes were filled with aCSF. Intracellular electrodes were filled with 3 M potassium acetate. Extracellular signals were amplified by a CyberAmp 320 (Molecular Devices, Palo Alto, CA, USA). Intracellular recordings were made using an Axoclamp 2A (Molecular Devices, Sunnyvale, CA, USA) microelectrode amplifier with 10× amplification. The amplified signals were digitized by an A/D converter card in an IBM-compatible computer, recorded by a Windows-based data acquisition program, and analyzed with a custom-made MATLAB program (MathWorks, Natick, MA, USA).

### Calcium images

Slices were stained with calcium dye to observe fluctuations in calcium transients during epileptiform activity. The staining method was based on a previous study [33]. The slices were placed on the porous membrane (pore size, 0.4 µm) of a Millicell-CM culture transwell. The dye solution was prepared with 48 µl dimethyl sulfoxide (DMSO; Sigma, St. Louis, MO, USA) and 2 µl F127 (10% in DMSO; Invitrogen) in 50 µg Fluo4-AM (Invitrogen). The dye solution (3 µl) was then dropped on the surface of the slice. The slices were incubated in the dark at 37°C for 30 min and then transferred to the MEA probe chamber. The tissue was illuminated with a 488 nm laser (Coherent, Santa Clara, CA, USA), and images were acquired at 2–5 Hz using an upright LSM510 META microscope (Zeiss, Oberkochen, Germany) equipped with a water immersion lens (63× magnification, 1.1 N.A; 20× magnification, 0.7 N.A) or a fluorescence stereomicroscope (Zeiss). The change in fluorescence over time was defined as 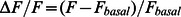, in which *F* was the fluorescence at any time point, and *F_basal_* was the baseline fluorescence averaged across the entire image for each cell. The correlations between calcium transients recorded in different cells were assessed by cross-correlation analysis.

### Potassium measurement

Borosilicate glass pipettes were silanized by *N,N*-dimethyltrimethylsilylamine (Fluka Chemicals) and back-filled with a potassium-selective liquid membrane (Fluka 60398). The rest of the pipette was then filled with 150 mM KCl [34], [35]. The electrodes were tested and calibrated with known changes in potassium concentration in the perfusion aCSF before and after the experiment. The data were obtained from electrodes that had the same sensitivity to potassium in both calibration trails.

### Western blot

To detect whether epileptiform activities change the expression pattern of Cx36 and Cx43, the mice were locally treated with 4-AP (2 µl, 20 µM) or saline (2 µl) in ACC. Mice were sacrificed after 5 h, the brains were quickly removed, and frontal lobe tissue was cut and homogenized with a Dounce tissue grinder (Fisher Scientific, Tustin, CA, USA). The tissue was washed twice in ice-cold wash buffer and incubated with extraction buffer I for 10 min at 4°C. Protease inhibitor (PI) mixture (Sigma, St. Louis, MO) was then added. The cells were then incubated with 1 ml of ice-cold extraction buffer II supplement with 10 µl PI for 30 min at 4°C. The cell suspension was centrifuged at 16,000×*g* for 15 min at 4°C. Supernatant that contained membrane proteins was collected. Protein concentrations were measured using a bicinchoninic acid protein assay kit (Pierce, Rockford, IL, USA). The protein complex was separated by sodium dodecyl sulfate-polyacrylamide gel electrophoresis (12.5%) and detected by Cx36 and Cx43 antibodies (1∶1000 dilution; Invitrogen). Each lane was loaded with 2 g of protein, and the amount of loading was checked by detecting tubulin. The dilutions of antibodies against enhanced green fluorescent protein and tubulin were 1∶10,000 and 1∶7,500, respectively. Labeled protein bands on the Western blots were visualized by enhanced chemiluminescence exposed on X-ray film (HyperFilm; Amersham Biosciences, Piscataway, NJ, USA).

### Drug application

The gap junction blockers octanol, carbenoxolone (CBX), and mefloquine (MFQ) and gap junction opener trimethylamine (TriMA) were purchased from Sigma. The GABA_A_ receptor antagonist bicuculline and 4-AP were purchased from Tocris Cookson (Ellisville, MO, USA). Stock solutions (4-AP, TriMA, and SPL in double-distilled water; bicuculline, CBX, and MFQ in dimethyl sulfoxide [DMSO]) were prepared, divided into small aliquots, and stored at −80°C. The aliquots were thawed on the experimental days, and all of the drugs were applied to the bath solutions according to their respective molar concentrations. For the local application of lidocaine or CBX, the drug solution was loaded in a syringe. The syringe was lowered onto the surface of the brain slice. The drug solution was then injected using a picoliter syringe pump at flow rate of 0.1 µl/min. The suction tube was placed near the site of drug administration to prevent diffusion [28].

### Data analysis

The data were analyzed using MC Rack software (Multi Channel systems) and the subroutines in the MATLAB program (MathWorks, Natick, MA, USA). To detect seizure-like events, we set three standard deviations of the noise level as the threshold. Seizure-like events were analyzed in their duration, frequency of occurrence and amplitude. The seizure-like activities were divided into ictal burst, tonic and clonic phase based on oscillation frequency recorded in field potential [36]. The amplitudes of the peaks during an oscillation event that surpassed this threshold were automatically detected by MC RACK software. The time-point of each peak that exceeded this threshold was also detected, and the duration of an oscillation event was measured by subtracting the time-point between the first and last peaks that surpassed the threshold. Two-dimensional current-source density (2D-CSD) profiles were calculated from the field potential profiles [28], [37] and color image plots were generated to facilitate visualization of the CSD profiles. Blue represents current sinks, and red represents current sources.

Field potential events were transformed to time frequency plots using Morlet continuous wavelet [38], [39]. The intensities in power were normalized to values between 0 and 1. Correlation of seizure-like activities recorded at different locations was evaluated by cross-correlation and coherence analysis. The channel where seizure-like activities initiate was selected as the reference for calculating the cross-correlation and coherence coefficients. The cross-coherence coefficient was calculated from the cross-spectral density [39], [40]. The computation was calculated according to the following formula:
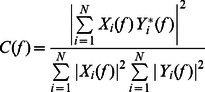
where Xi (f) and Yi (f) are the Fourier transforms of the signals x and y for the i data segment at frequency f, and * indicates the complex conjugate. The computations were performed by using the magnitude-squared coherence function (MATLAB) based on Welch's averaged periodogram method (non-overlapping 0.5 s time window, frequency resolution of 2 Hz).

The data are expressed as mean ± SE. Statistical analyses were performed with Systat (SPSS) and Microsoft Excel software using Student's *t*-test. One-way or two-way ANOVA was used to analyze the effects of gap junction blockers on the duration, amplitude, incidence of seizure-like activities. Tukey's post hoc test was used to detect the sources of group differences revealed by the ANOVA. Measurements in the text are expressed as mean ± SE, and *n* indicates the number of slices or neurons studied. The results were considered significant at *p*≤0.05.

## Results

### Cingulate seizure induction and effects of gap junctions

**Figure 1:**
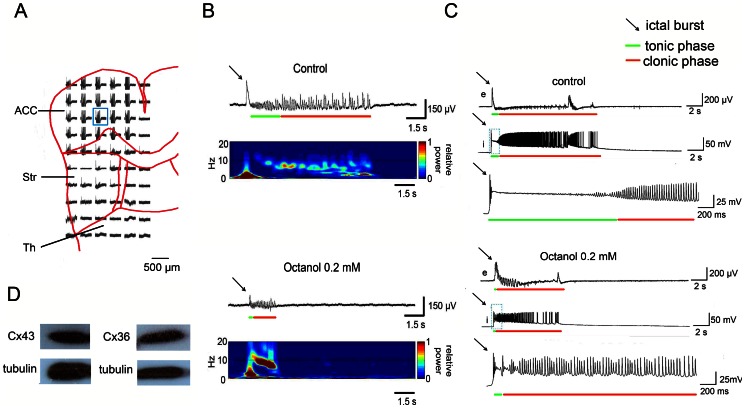
Influence of gap junctions on cingulate seizures. (A) Position of the MEA against a MT-ACC slice. Ensemble seizure-like activity induced by 4-AP and bicuculline is shown. The channel surrounded by the blue square is magnified in C. (B) Simultaneous extracellular (e) and intracellular (i) recording showed that the tonic phase corresponded to a depolarization phase in the intracellular recording, whereas the clonic phase was correlated with bursting activity. After the application of octanol, the depolarization phase and bursting activity were shortened. (C) Typical example of changes in seizure-like activity after the application of octanol. 4-AP- and bicuculline-induced seizure-like activity is composed of ictal onset (arrow), a tonic phase (green line), and a clonic phase (red line). The application of 0.2 mM octanol shortened the tonic and clonic phases. (D) Western blot showed that both glial gap junction Cx43 and neuronal-specific gap junction Cx36 are expressed in the cingulate cortex.

Drug-resistant seizure-like activity was induced by co-administration of 4-AP (250 µM) and bicuculline (5 µM). Seizure-like activity appeared after 10 min of drug application. Ensembles of seizure-like activity are shown in Fig. 1A. Seizure-like activity was most prominent in cingulate regions. Our previous research showed that ictal bursts first appeared in the cortex and then propagated to the striatum and thalamus [28]. The analysis of the occurrence of seizure onset confirmed our earlier observations. Seizure-like activity induced by 4-AP and bicuculline were divided into ictal onset (Fig. 1B, arrow), a tonic phase (Fig. 1B, green line), and a clonic phase (Fig. 1B, red line) based on frequency evolution shown by wavelet transformed from field potential recording [41]. Typical traces were taken from the ensemble recording (Fig. 1A, blue square). A wavelet transformation showed that the dominant frequency of seizure-like activity was in the delta and theta ranges (4–8 Hz). Application of the gap junction blocker octanol (0.2 mM) significantly shortened both the tonic and clonic phases and decreased the amplitude of seizure-like activity (Fig. 1B, lower panel). Paired extra- and intracellular recordings showed that the tonic phase corresponded to the depolarization of pyramidal neurons, and the clonic phase corresponded to the bursting activity of pyramidal neurons (Fig. 1C). The application of octanol significantly decreased the duration of the depolarization state and bursting activity of pyramidal neurons during the clonic phase (Fig. 1C, n = 7). The average resting membrane potential was −68.4±0.91. After 4-AP and bicuculline application, the membrane potential shift to −63.2±1.45 and −63.8±1.26 after gap junction blocker application. The expression of gap junctions in the frontal lobe was confirmed by Western blot. The neuronal-specific gap junction connexin 36 (Cx36) and glial gap junction connexin 43 (Cx43) were expressed in the frontal lobe region (Fig. 1D). To test whether the expression of gap junctions was altered by seizure induction, the mice were sacrificed after 5 h of 4-AP or saline treatment in the ACC. The results showed that the expression levels of Cx36 and Cx43 were not significantly different between the control and 4-AP groups (*n* = 3, *p* = 0.47, *t*-test), indicating that the expression of gap junctions was not influenced by seizure induction.

### Gap junction blockers attenuate seizure-like activity

**Figure 2:**
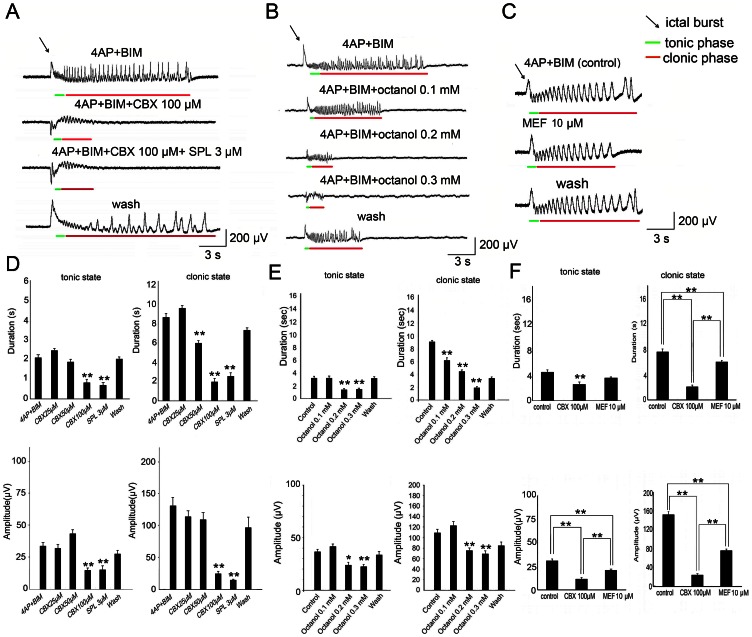
Effect of different gap junction uncouplers on seizure-like activity in thalamocingulate slice. (A) Representative traces of 4-AP+bicuculline-induced seizure-like activity. The application of 100 µM CBX significantly decreased the amplitude and duration of seizure-like activity in both the tonic and clonic phases. The application of 3 µM SPL did not change the effect of CBX. Brain slice washes with 4-AP and bicuculline partially restored seizure-like activity. (B) The statistical results showed that the duration of the clonic phase was significantly shortened after the application of 50 µM CBX. The duration and amplitude of both the tonic and clonic phases of seizure-like activity were significantly decreased after 100 µM CBX application. (C) Another gap junction uncoupler, octanol, also effectively decreased seizure-like activity. (D) The statistical results showed that 0.1 mM octanol was sufficient to significantly shorten the duration of the clonic phase of seizure-like activity. The duration and amplitude of the tonic phase was shortened by the application of 0.2 mM octanol. The amplitude of the clonic phase was also shortened by 0.2 mM octanol application. (E) The neuronal-specific gap junction blocker MFQ was tested and also modulated seizure-like activity. (F) The statistical results showed that 10 µM MFQ did not change the duration of the tonic phase of seizure-like activity. However, 10 µM MFQ significantly decreased the amplitude of the tonic phase. The clonic phase was sensitive to MFQ application. MFQ (10 µM) significantly decreased the amplitude and duration of the clonic phase. CBX was significantly more effective in suppressing seizure-like activity compared with the MFQ treatment groups.

The effects of different gap junction blockers were tested on cingulate seizure-like activity. The maximal inhibitory effect of CBX appeared after 30 min application. Thus, all of the comparisons were performed after 30 min of CBX application. Typical traces were selected from the ensemble recording in the cingulate cortex. Low concentrations of gap junction blockers (50 µM CBX and 0.1 mM octanol) significantly shortened the duration of the clonic phase (Fig. 2A, B, D, and E; *n* = 7, *p<0.05, *t*-test), but no significant effect was observed during the tonic phase. High concentrations of the gap junction blockers (100 µM CBX and 0.2 mM octanol) effectively shortened the duration of both the tonic and clonic phases. The amplitudes of both phases were also significantly decreased after the application of high concentrations of the gap junction blockers (Fig. 2A, B, D, and E; *n* = 7, *p<0.05, *t*-test). To exclude the possibility that CBX might also act as a mineralocorticoid receptor agonist, the mineralocorticoid antagonist spironolactone (SPL) was also applied [42]. The effects of co-administration of SPL (3 µM) and CBX (100 µM) on seizure-like activity was not significantly different from the application of 100 µM CBX alone (Fig. 2A and D; *n* = 6, *p* = 0.57, *t*-test), indicating that the inhibitory effect of CBX did not occur through the activation of mineralocorticoid receptors. Washes with 4-AP and bicuculline in aCSF for 2 h partially restored seizure-like activity in 32.7% (17 of 32) of the slices. The incidence of seizure-like activities was not significantly altered by the application of 100 µM CBX or 0.2 mM octanol. The inter-event interval of seizure like activities was 145.2±21.4 s in control (4-AP+bicuculline) group, 139.8±22.4 s in CBX group and 152.3±25.1 s in octanol group.

The effects of the neuronal-specific gap junction blocker MFQ were also tested. Typical traces are shown in Fig. 2C. The application of MFQ (10 µM) significantly lowered the amplitude and duration of seizure-like activity in the clonic state (Fig. 2F; *n* = 6, *p<0.05, *t*-test). No significant difference was found during MFQ treatment in the tonic state. The results showed that MFQ significantly decreased the amplitude and duration of seizure-like activity, but the effects of CBX were more potent (Fig. 2F; *n* = 6, *p<0.05, *t*-test). This result showed that gap junctions in both neurons and glial cells played important roles in cingulate seizure-like activity.

### The effect of gap junction blockers acts on cortex

**Figure 3:**
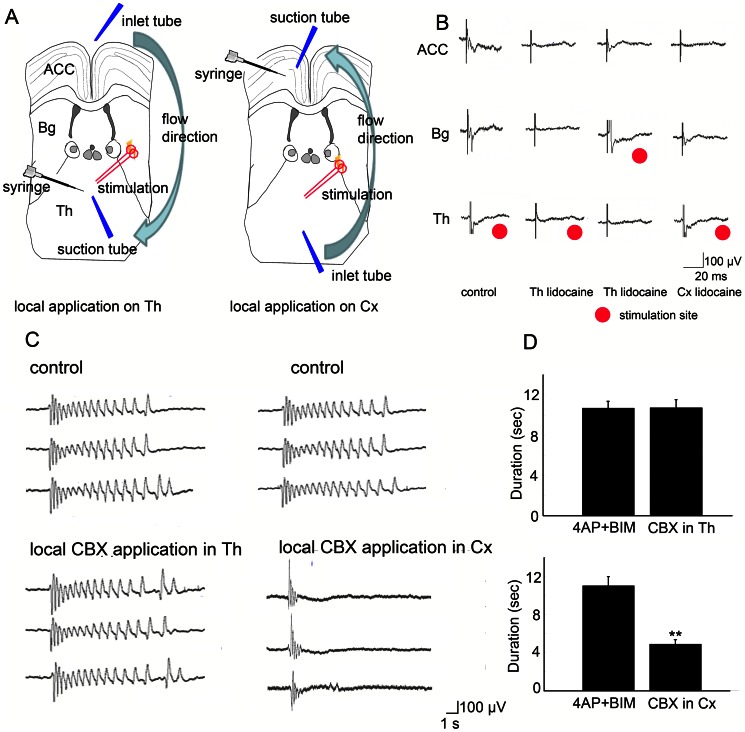
The gap junction blocker CBX acts in the cortex. (A) Diagram that indicates the orientation of the stimulation electrode, syringe for drug application, and suction tube against the brain slice. Local application of the drug in the thalamus is shown in the left panel. Local application of the drug in the cingulate cortex is shown in the right panel. (B) The thalamus-evoked cortical response was diminished after lidocaine application in the thalamus. The cortical response was still evoked by stimulation of the basal ganglia (Bg). This indicates that the effect of lidocaine was restricted to the thalamus. When lidocaine was applied to the cortex, electrical evoked responses from the thalamus propagated to the basal ganglia but cortex. (C) Example sweeps from three electrodes showed cortical seizure-like activity. Local application of CBX in the thalamus did not influence cortical seizures, whereas local CBX application in the cortex shortened the duration of seizure-like activity. (D) The statistical results showed that local CBX application in the cortex significantly shortened the duration of seizure-like activity.

Because bath application of gap junction blockers will block gap junctions in both the cingulate cortex and thalamus, we tested the relative importance of gap junctions in these two regions in the modulation of seizure-like activity. We previously tested the local contribution of the thalamus in modulating cortical seizure-like activity (Chang et al., 2011). The same procedure was applied to test the relative contribution of gap junctions in the cortex and thalamus. We first used lidocaine to test the diffusion range of local drug application in our system. The experimental setup is depicted in Fig. 3A. The suction tube was placed near the drug-delivery syringe. The stimulation electrode was placed in the thalamus. The thalamus-evoked cortical response was diminished by 1 µl lidocaine application in the thalamus. Moving the electrode to the basal ganglia still evoked a cortical response, indicating that the diffusion range of the drug was confined to the thalamic region. Thalamic stimulation did not evoke a cortical response when lidocaine was locally applied in the cortex. The response was still present in the basal ganglia, indicating that the drug diffusion range was restricted to the cortical region (Fig. 3B). When 100 µM CBX was applied to the thalamus, seizure-like activity was not significantly altered. However, when 100 µM CBX was applied to the cortex, seizure-like activity was transiently suppressed (Fig. 3C, D; *n* = 5; *p<0.05, *t*-test). The effect of local CBX application on cortical seizures was the same as CBX when it was previously applied in the perfusion chamber. These results indicate that the major action of the gap junction blocker was in the cortex rather than in the thalamus in our MT-ACC brain slice preparation.

### Gap junction opener enhances seizure-like activities

**Figure 4:**
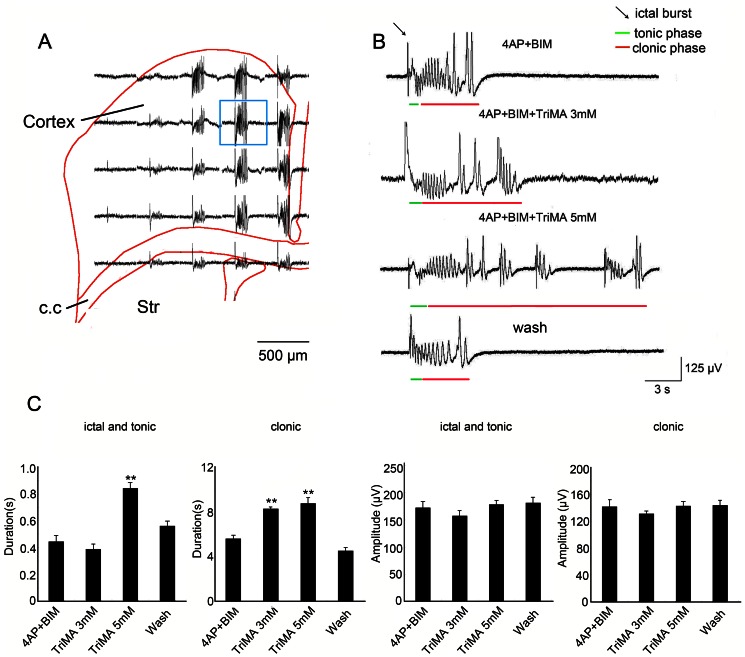
Gap junction opener TriMA enhances seizure-like activity. (A) Typical example of ensemble seizure-like activity. The channel surrounded by the blue square is magnified in B. (B) Typical seizure-like activity showed that the application of 5 mM TriMA enhanced the clonic phase of seizure-like activity (red lines). The effects of TriMA were reversible. Brain slice washes with 4-AP and bicuculline restored seizure-like activity to original levels. (C) The statistical results showed that the amplitude of seizure-like activity was not changed by TriMA in either the ictal-tonic or clonic phase. The duration of the ictal-tonic phase was significantly enhanced after 5 mM TriMA application. The clonic phase was enhanced by 3 mM TriMA application. Cx, cortex; Str, striatum; Th, thalamus.

If the clonic phase of seizure-like activity is sensitive to gap junction blockade, then increasing the opening probability of gap junctions should facilitate the appearance of clonic discharges. Therefore, the effect of a gap junction opener, TriMA, on seizure-like activity was tested. Typical ensembles of seizure-like activity induced by 4-AP and bicuculline are shown in Fig. 4A. The channel surrounded by a blue square was selected for subsequent presentation in Fig. 4B. TriMA application enhanced the duration of the clonic phase (Fig. 4B, red lines) and tonic phase (Fig. 4B, green line) of seizure-like activity (Fig. 4B, red lines). The effect of TriMA could be washed out with 4-AP and bicuculline (Fig. 4B). The duration of the tonic phase was significantly prolonged after the application of 5 mM TriMA (Fig. 4B and C; *n* = 5, *p<0.05, *t*-test). The duration of the clonic phase was significantly enhanced by a lower concentration of TriMA (3 mM; Fig. 4B and C; *n* = 5, *p<0.05, *t*-test). However, the amplitudes of the tonic and clonic phases of seizure-like activity were not significantly altered by the application of TriMA (Fig. 4C; *n* = 5, ANOVA). The incidence of seizure-like activities was not significantly altered by the application of 5 mM TriMA. The inter-event interval of seizure like activities was 140.8±19.2 s in control (4-AP+bicuculline) group, and 133.5±12.2 s in TriMA group.

### Gap junctions are involved in the maintenance of seizure-like activity

**Figure 5:**
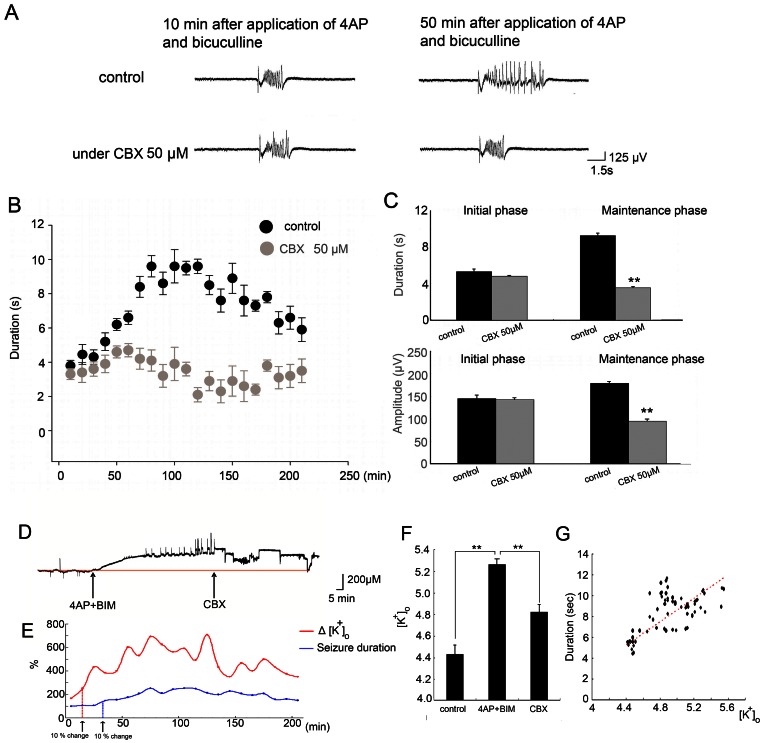
Gap junctions are involved in the maintenance of seizure-like activity. (A) In the 4-AP and bicuculline control group, seizure-like activity began to appear after 10 min and reached a maximum after 80 min (upper panel). The application of 100 µM CBX 30 min prior to the application of 4-AP and bicuculline did not delay the onset of seizure-like activity. However, in the CBX group, seizure-like activity was not potentiated (lower panel). (B) Statistical results of the changes in the duration and amplitude of seizure-like activity over time. The duration and amplitude in the 4-AP and bicuculline control group were significantly greater than in the CBX group after 50 min of seizure induction. (C) The statistical results showed that CBX did not significantly influence the initial phase, but CBX could significantly decreased the duration and amplitude of seizure-like activity in the maintenance phase. (D) Time-lapse recording of potassium concentration. (E) Development of seizure-like activity. The potassium concentration increased before seizure-like activity turned into a longer duration. (F) Statistical results of time-lapse recording of potassium concentration. The application of 4-AP and bicuculline significantly increased potassium concentration, whereas the application of CBX significantly decreased it. (G) The duration of seizure-like activity was positively correlated with the concentration of extracellular potassium ions.

To test whether gap junctions are involved in the induction and maintenance of seizure-like activity in the cingulate cortex, CBX (50 µM) was applied 30 min prior to the application of 4-AP and bicuculline. Seizure-like activity began to appear after 10 min of 4-AP and bicuculline application, and the maximal drug effects lasted from 50 min to 3 h. Typical seizure-like activity selected from ensemble recordings after 10 and 50 min of 4-AP and bicuculline application are shown in Fig. 5A. The application of CBX (50 µM) did not influence the time of onset of seizure-like activity. Seizure-like activity also appeared after 10 min of 4-AP and bicuculline application under the application of CBX. The amplitude and duration were not significantly different between the control and CBX groups in the initial phase (Fig. 5A, left panel, and C; *n* = 5, *p<0.05, *t*-test). However, in the CBX application groups, seizure-like activity was not potentiated. The amplitude and duration of seizure-like activity were significantly smaller in the CBX groups than in the control groups after 50 min of 4-AP and bicuculline application (Fig. 5A and C; *n* = 5, *p<0.05, *t*-test). Potassium was measured from the beginning of the experiment, and typical traces of potassium recordings are shown in Fig. 5D. Notice that the potassium concentration increased after the application of 4-AP and bicuculline. Potassium concentrations decreased after the application of CBX. The average concentration of potassium ions during the control period was 4.4±0.56 mM. After the application of 4-AP and bicuculline, the potassium concentration reached 5.1±0.82 mM. The accumulation of potassium is prior to the development of seizure-like activities into longer duration, extracellular potassium concentration surpass 10% of it's maximum concentration before seizure-like activity developed. (Fig. 5E, arrows). The results showed that 4-AP and bicuculline application significantly increased potassium concentrations, whereas CBX application significantly decreased potassium concentrations (Fig. 5F; *n* = 5, *p<0.05, *t*-test). The extracellular concentration of potassium was positively correlated with the duration of seizure-like activity (Fig. 4G; *r^2^* = 0.515, *p<0.01, linear regression).

### Gap junctions are involved in the theta oscillation of seizure-like activity

**Figure 6:**
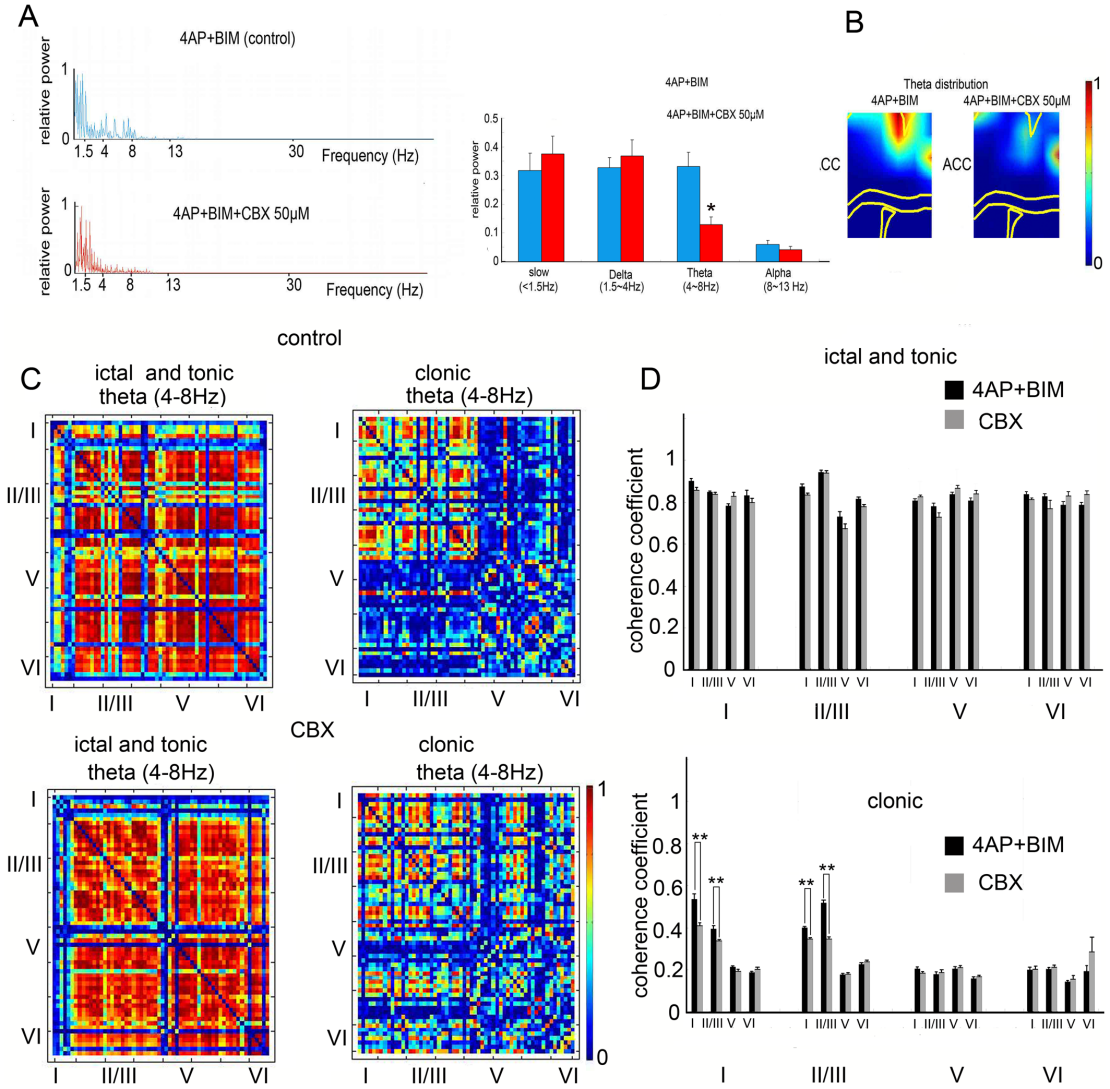
Gap junctions decrease theta range oscillation. (A) The frequency analysis showed that the frequency of seizure-like activity predominantly occurred in the delta (1.5–4 Hz) and theta (4–8) ranges. The application of CBX attenuated seizure-like activity in the theta range. The statistical results showed that CBX significantly lowered θ range oscillation. (B) Average color plot of theta distribution during seizure-like activity and after the application of CBX. The application of CBX significantly decreased the distribution area of theta oscillations. (C) The coherence coefficient map of theta oscillation showed that the correlation between each channel was strong, even after CBX application, during the ictal and tonic phase. In the clonic phase, the correlation between channels in the superficial layer deceased after the application of CBX. (D) The statistical results showed that the coherence coefficient between layers was not significantly different between the control and CBX groups during the ictal-tonic phase. After the application of CBX, the theta correlation was significantly decreased in the superficial layer. ACC, anterior cingulate cortex; cc, corpus callosum; Bg, basal ganglia; Th, thalamus.

Gap junctions are involved in synchronizing large neuronal ensembles at different frequency bands and have been implicated in different physiological and pathophysiological processes [43], [44]. To determine in which frequency bands gap junctions are involved during seizure-like activity in the cingulate cortex, we compared the fast-Fourier transformation of seizure-like activity before and after CBX application. Fast-Fourier transformations of typical seizure-like activity are shown in Fig. 6A. Notice that application of CBX significantly lowered theta band oscillation (Fig. 6A; *n* = 6, *p<0.05, *t*-test). The distribution profiles of the theta frequency were averaged and are shown in Fig. 6B. Notice that theta oscillations were distributed through the superficial layer of the cortex before the application of the gap junction blocker. Application of the gap junction blocker attenuated the theta frequency in the superficial layer of the ACC. Coherence coefficients between each layer in the theta frequency were analyzed. The coherence coefficients between each layer were not significantly different between the control and CBX treatment groups during the ictal and tonic phases of seizure-like activity (Fig. 6C and D; *n* = 6, *p<0.05, *t*-test). However, the coherence coefficient in the superficial layer was lowered after CBX application during the clonic phase (Fig. 6C). The coherence coefficient was significantly lowered within the layer I channels and between the layer I and layer II channels after CBX application (Fig. 6D; *n* = 6, *p<0.05, *t*-test).

### Gap junctions alter the synchrony of seizure-like activity

**Figure 7:**
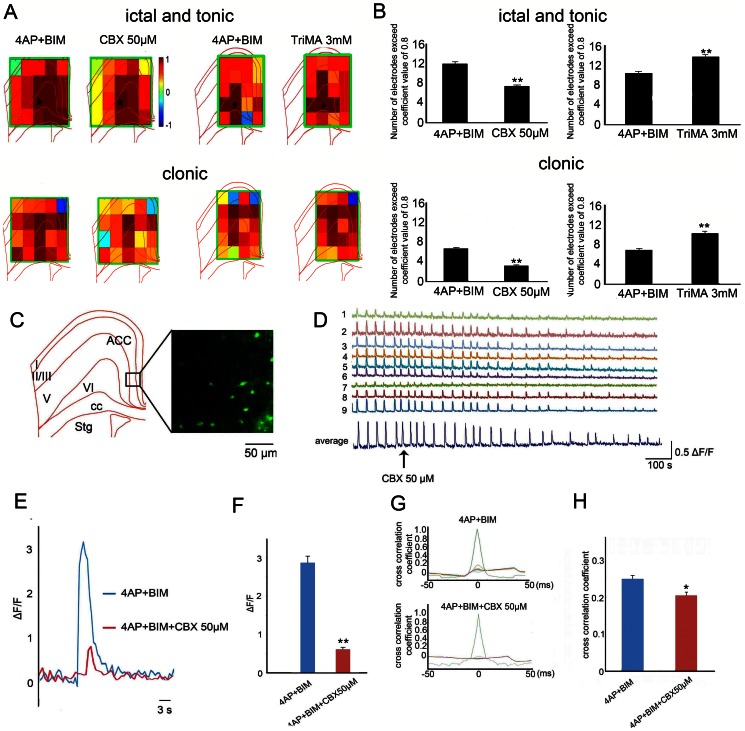
Gap junction blocker attenuates cross-correlation and calcium transient of seizure-like activity. (A) Average cross-correlation coefficient pseudocolor map between control, CBX, and TriMA. (B) The statistical results showed that the cross-correlation coefficient of seizure-like activity was significantly decreased after the application of CBX and increased after the application of 3 mM TriMA in both the ictal-tonic and clonic phases. (C) Calcium imaging focused on the most frequent seizure-initiation onset (black square). Calcium transients were induced by 4-AP and bicuculline. (D) Individual and average traces of calcium transients. Notice that the amplitude of the calcium transient was gradually attenuated after the application of CBX. (E) Changes in fluorescence were compared. The amplitude of the calcium transient was significantly larger before applying CBX. (F) The statistical results showed that the calcium transients were significantly decreased after the application of 50 µM CBX. (G) The synchronization index of the calcium transients between each cell was decreased after the application of CBX. (H) The statistical results showed that CBX significantly decreased the synchronization index of the calcium transients. cc, corpus callosum; Cx, cortex; Str, striatum; Th, thalamus.

To estimate the changes in the temporal dimension of ictal-tonic and clonic activity-mediated functional coupling in response to the application of gap junction modulators, cross-correlation coefficients for seizure activity in the cortex (Fig. 7A, green square) were calculated relative to one reference channel where the seizure began (Fig. 7A; the asterisk represents the reference channel). The application of 50 µM CBX significantly lowered the cross-correlation coefficient in both the ictal-tonic and clonic phases (Fig. 7B; *n* = 6, *p<0.05, *t*-test). To test whether TriMA enhances synchronicity between different cortical regions, cross-correlation coefficients between each channel covered in the cortex were compared before and after the application of TriMA. The application of 3 mM TriMA significantly increased the cross-correlation coefficients between each channel in both the ictal-tonic and clonic phases (Fig. 7B; *n* = 5, *p<0.05, *t*-test). To test whether gap junction blockers influence synchronicity between different neurons, cross-correlation coefficients between calcium transients between cells were analyzed. The calcium transients induced by 4-AP and bicuculline were recorded with and without application of CBX. Our previous study showed that seizure-like activity in calcium dye-labeled cells was mostly initiated in layers II/III and V [28]. Therefore, calcium imaging was focused on these layers (Fig. 7C, black box) to study the role of gap junctions in calcium transient activity. Fluo-4-labeled cells are shown in Fig. 7C (right panel). Temporal changes in the calcium transients obtained from different cells and averaged traces are shown in Fig. 7D. Notice that the calcium transient began to decrease after the application of CBX (50 µM). The average calcium transients before and after the application of CBX are shown in Fig. 7D. The calcium transients significantly decreased after CBX application (Fig. 7E and F; *n* = 6, *p<0.05, *t*-test). The application of CBX also significantly decreased synchronicity between different cells (Fig. 7G and H; *n* = 6, *p<0.05, *t*-test).

### Modulatory effect of thalamic inputs on seizure-like activity is partially mediated by gap junctions

**Figure 8:**
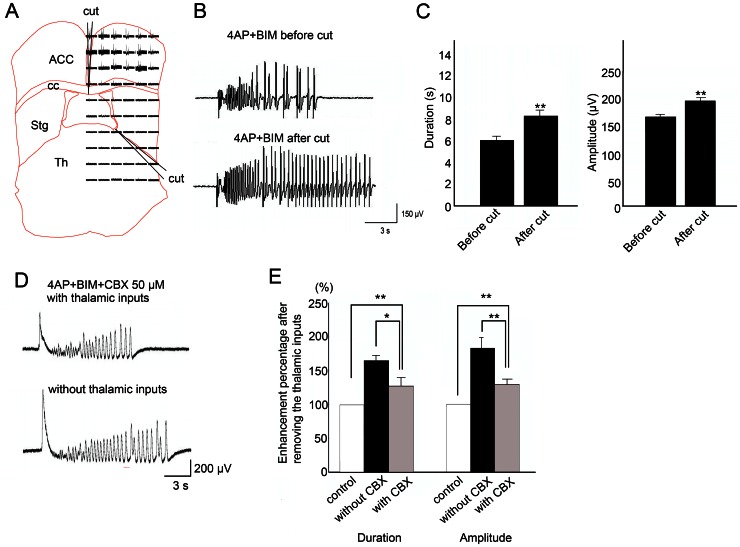
Modulatory effect of thalamic inputs on seizure-like activity is partially mediated by gap junctions. (A) The corpus callosum was severed to prevent synchronization between the right and left cortices. MT inputs were removed by cutting. The ensembles of seizure-like activity recorded by the MEA are shown. A typical example shows that removing the thalamic inputs increased the duration and amplitude of seizure-like activity during the application of 4-AP, BIM, and CBX (50 µM). (C) The statistical results showed that the duration and amplitude of seizure-like activity were significantly increased by the application of CBX. (D) Typical example of seizure-like activity induced by the application of 4-AP and bicuculline. Notice that the duration and amplitude of seizure-like activity increased after removing the thalamic inputs. (E) The statistical results showed that the application of CBX significantly attenuated the enhancing effect of removing the thalamic inputs on the duration and amplitude of seizure-like activity. cc, corpus callosum; Cx, cortex; Str, striatum; Th, thalamus.

To investigate the involvement of gap junctions in the thalamocingulate system during seizure-like activity, we compared seizure-like activity with and without thalamic inputs under the influence of the gap junction blocker CBX. To prevent the propagation of seizure-like activity from the contralateral side, the corpus callosum was cut in the junction between the left and right cortex as described previously [28]. The thalamic inputs were first severed at the junction between the basal ganglia and thalamus to test whether thalamic inputs influence seizure-like activity (Fig. 8A). We previously showed that modulation by local perfusion of lidocaine is equally effective as thalamic severing [28]. The duration and amplitude of seizure-like activity increased after removing the thalamic inputs when incubated in 4-AP and bicuculline (Fig. 8B). Notice that the amplitude and duration of seizure-like activity increased after removing the thalamic inputs. The results showed that removing the thalamic inputs significantly enhanced the duration and amplitude of seizure-like activity (Fig. 8C; *n* = 5, *p<0.05, *t*-test). This result confirmed our previous observation [28]. Typical examples selected from ensemble recordings before and after removing the thalamic inputs under the application of 50 µM CBX are shown in Fig. 8D. The enhancing effect of removing the thalamic inputs on seizure-like activity was significantly less in the CBX treatment groups (Fig. 8E; *n* = 5, *p<0.05, *t*-test). Removing thalamic inputs with or without CBX treatment did not significantly change the incidence of seizure-like activities. The inter-event interval of seizure like activities was 145.5±15.4 s in control (4-AP+bicuculline) group, and 124.4±15.1 s in thalamic removing under CBX group, and 133.4±18.2 s in thalamic removing without CBX group. When the MT-ACC slice was only incubated in 4-AP and bicuculline, the enhancing effect of removing the thalamic inputs was greater than treatment with CBX. The duration and amplitude of cortical seizure-like activity were more enhanced after removing the thalamic inputs in the 4-AP and bicuculline treatment groups. The enhancing effects on seizure-like activities after removal of thalamic inputs was partially repressed by the application of CBX, indicated that the thalamic inputs modulates seizure-like activities partially through the regulation of gap junctions.

### Gap junction blockers modulate the spatiotemporal propagation pattern of seizure-like activity

**Figure 9:**
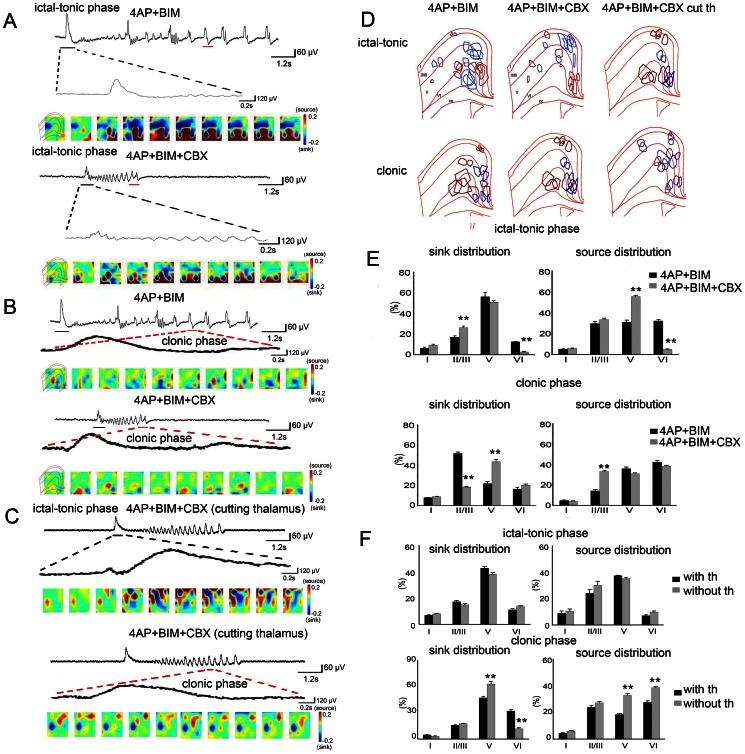
Gap junction blocker modulates the spatiotemporal propagation pattern of seizure-like activity. (A) Typical seizure-like activity before and after the application of CBX. The black and red lines indicate the segments are enlarged below. (B) The ictal-tonic phase of seizure-like activity (black line in A) was magnified. The spatiotemporal change in 2D-CSD was plotted according to different time-points. Notice that sink signals (blue) were more concentrated in layer V during the ictal-tonic phase after the application of 50 µM CBX. Sink signals were enhanced in the superficial layer. During the clonic phase, sink signals were enhanced in layer V and decreased in layer II/III after the application of CBX. (C) A typical seizure-like event was divided into ictal-tonic and clonic phases. The 2D-CSD pseudocolor map shows the spatiotemporal evolution of seizure-like activity without thalamic inputs under the influence of CBX. (D) The patterns of sink-source distributions from five different slices were superimposed. (E) The statistical results showed that sink signals significantly increased in layer II/III and decreased in layer V after the application of CBX during the ictal-tonic phase. Source signals significantly increased in layer V and decreased in layer VI. During the clonic phase, sink signals significantly increased in layer V and decreased in layer II/III, whereas source signals significantly increased in layer II/III after CBX application. (F) The statistical results showed that the sink signals were located in layer V when the thalamic inputs were intact, whereas source signals were also located in layer V during the ictal-tonic phase. Removing the thalamic inputs did not significantly change the sink-source distribution during the ictal-tonic phase. In the clonic phase, removing the thalamic inputs significantly increased sink signals in layer V and decreased them in layer VI, whereas source signals were enhanced in layers V and VI.

Two-dimensional current source density was used to determine whether the spatiotemporal patterns of seizure-like activity are influenced by gap junction blockers. A typical example of seizure-like activity is shown in Fig. 9A. Black and red lines beneath the traces indicate the time-point for the 2D-CSD profile analysis in the ictal-tonic and clonic phases, respectively. During the ictal-tonic phase, the sink signals (red) appeared in the deep layers. After the application of 50 µM CBX, the source signal (blue) shifted to the superficial layer (Fig. 9A). Superimposing the 2D-CSD analysis of the ictal-tonic phase of six samples indicated that the sink currents (blue) aggregated in layers V and VI, whereas the source current (red) occurred more frequently in the superficial layer (Fig. 9D). After the application of the gap junction blocker, the sink currents shifted to the superficial layers, and the sink current occurred more frequently in layer V. The results showed that the sink signal was significantly enhanced in layer II/III after CBX application and decreased in layer VI. The source signal increased in layer V and decreased in layer VI (Fig. 9E; *n* = 6, *p<0.05, *t*-test). During the clonic phase, the sink signals were more concentrated in the superficial layer, whereas the source signals were in deeper layers (Fig. 9B and D). After the application of CBX, the sink signal shifted to layer V, and the source signal shifted to layer II/III. The results showed that the sink signals were significantly enhanced in layer V and decreased in layer II/III after the application of CBX, whereas the source signals were significantly enhanced in layer II/III (Fig. 9E; *n* = 6, *p<0.05, *t*-test).

We examined the extent to which the spatiotemporal profile of cingulate seizure activity is influenced by thalamic inputs through alterations in gap junctions. The thalamic inputs were removed under the influence of the gap junction blocker CBX (50 µM). Seizure-like activity in the ACC was compared between groups with and without thalamic inputs. The typical spatiotemporal evolution of 2D-CSD is shown in Fig. 8C. Under the influence of CBX, during the ictal-tonic phase, the sink-source signals were mainly distributed in layer V (Fig. 9C and D). The 2D-CSD sink and source signals were superimposed from the five different brain slices shown in Fig. 8D. Removing the thalamic inputs did not significantly change the sink-source distribution during the ictal-tonic phase (Fig. 9D and F; *n* = 5, *p<0.05, *t*-test). During the clonic phase, the sink-source signals were also distributed in the deep layers, but removing the thalamic inputs significantly enhanced the sink signal in layer V and decreased it in layer VI. The source signals were significantly enhanced in deeper layers after removing the thalamic inputs (Fig. 9D and F; *n* = 5, *p<0.05, *t*-test).

## Discussion

Our results showed that drugs that block gap junctions are able to decrease the clonic phase of seizure-like activity in the cingulate cortex. Conversely, the application of the gap junction opener TriMA significantly increased the clonic phase of seizure-like activity. Comparisons of seizure-like activity among the control, gap junction blocker, and gap junction opener groups revealed the following. First, the gap junction blockers CBX and octanol significantly decreased seizure-like activity, whereas the gap junction opener TriMA enhanced seizure-like activity. The neuronal-specific gap junction blocker also attenuated seizure-like activity, but its potency was less than the broad-spectrum gap junction blockers. Second, the spatiotemporal 2D-CSD profile revealed a pronounced sink signal shift to the superficial layer after the application of CBX. Third, the theta frequency of seizure-like activity was significantly reduced during CBX application. Fourth, calcium imaging demonstrated that calcium-mediated bursting was reduced after CBX application. The synchronicity between each cell also decreased. Fifth, the modulatory effect of thalamic inputs on seizure-like activity occurred partially through gap junctions. Thus, gap junctions play an important role in synchronizing seizure-like activity and mediating thalamic modulatory effects.

Previous studies showed that the neuronal Cx36 marker is evident in all neocortical layers where cell bodies were located. The expression pattern of Cx36 is layer- and region-specific. The expression is modest in the piriform cortex but increases dorsally to very high levels in primary motor and cingulate areas. Cx36 expression was not found in thalamic regions, with the exception of the reticular thalamus [45]. Other studies that used lacZ to determine the expression of Cx36 in transgenic mice also found that Cx36 is strongly expressed in the cingulate gyrus [46].

Our results showed that gap junctions are significantly involved in the regulation of the clonic phase of seizure-like activity in the cingulate cortex. We found that ictal bursts and the tonic phase of seizure-like activity, clinically manifested as the tonic phase of a generalized seizure [47], were not influenced by gap junction openers or blockers, whereas the subsequently appearing clonic phase was enhanced by the application of the gap junction opener and inhibited by the gap junction blocker. The synchronization and propagating mechanism of ictal bursts and the tonic phase of seizure-like activity induced by 4-AP and bicuculline depend on synaptic transmission mediated by both 2-amino-3-(3-hydroxy-5-methyl-isoxazol-4-yl) propanoic acid and *N*-methyl-D-aspartate receptors [48]–[50], and gap junctions are not involved in synaptically synchronized primary bursting activity [48].

The involvement of gap junctions in the maintenance of seizure-like activity was also demonstrated by application of the gap junction blocker 30 min prior to the application of 4-AP and bicuculline. Our results showed that application of the gap junction blocker did not influence the induction of seizure-like activity. 4-AP and bicuculline induced seizure-like activity, reaching maximal responses after 50 min of application. Within 50 min, the amplitude and duration of seizure-like activity were not significantly different between the CBX and 4-AP+bicuculline groups, indicating that gap junctions are not involved in the induction stage of seizure-like activity. The significant decrease in the duration of seizure-like activity induced by CBX may be mediated by the depression of synchronization between neurons [5]. Although CBX is also a mineralocorticoid agonist, such receptors are not involved in seizure-like activity induced by 4-AP or a Mg^2+^-free solution [4]. We also applied the mineralocorticoid antagonist SPL to exclude the possibility that CBX might also act on this receptor.

CBX applied in this experiment began to influence the patterns of seizure-like activities significantly after 30 min of application. The delays of action of these gap junction blockers on seizure-like activities may be attributed to their effects on gap junctions rather than their non-specific effects on calcium channel, AMPA and NMDA receptor [51]–[53]. With intracellular recordings, we found that the application of octanol did not alter the resting membrane potential. Previous studies also showed that CBX, octanol, and MFQ did not influence neuronal excitability [48], [54], [55]. We have also found that CBX did not significantly influence the seizure-like activities during the first 30 min of application, supporting the view that the findings reported here in slices did not reflect some unspecific change in neuronal excitability as reported in cultured neurons [52].

Mefloquine, a derivative of quinine is a potent gap junction blocker. MFQ could completely block Cx36 and Cx50 at 3 µM concentration [56]. Cx50 are expressed in lens fiber cells of human and mouse eyes [57]. The effect of MFQ on Cx50 could be excluded in the brain slice preparation. Higher concentration of MFQ (30 µM) could also block Cx43 [56]. The concentration of MFQ we applied was 10 µM to prevent nonspecific closure of other types of gap junctions. However, we cannot rule out the possibility that the effects reported here were partially mediated by an action of MFQ on pannexins [58]. A previous study showed that quinine could completely block GABAergic ictal-like events in rat hippocampal slices [59]. Quinine also suppressed spontaneous seizure-like activity without decreasing neuronal excitability in low-Ca^2+^ and high-K^+^ seizure models [60]. Moreover, mefloquine inhibited Cx36-mediated cortical spreading depression in a rat neocortical slice model [61]. Altogether, experimental evidences suggest that the neuronal-specific gap junction blocker mefloquine may block GABAergic synchronization.

To distinguish between the neuronal and glial gap junction effects on seizure activity, we tested broad-spectrum (CBX) and neuronal-specific (MFQ) gap junction blockers. We found that CBX has higher efficiency in blocking seizure-like activity compared with MFQ. This result suggests that both neurons and glial cells play an important role in seizures. The pharmacological results showed that both neuronal and glial gap junctions are involved in the maintenance phase. Both broad-spectrum and neuronal-specific gap junction blockers suppressed seizure-like activity. Previous studies indicated that 4-AP induce seizure-like activities were markedly attenuated in Cx36 knockout mice [62]. These results indicate that both neurons and glial cells play important roles in seizure regulation.

A recent study demonstrated the important role of glial cells in seizures [63]. Astrogliosis is often found postmortem and in surgical resections of brains obtained from patients with chronic temporal lobe epilepsy [64]. Glial cell networks are involved in the generation and maintenance of epileptiform activity [65]. The hyperactivity of neurons during epileptiform activity could increase extracellular potassium ions, which consequently leads to a decrease in the volume of the extracellular space. Astrocytes are important for buffering potassium concentrations in the extracellular milieu around neurons, and previous studies showed that spatial potassium buffering ability is impaired in Cx30/Cx43 double knockout mice [66]. An impaired capacity of potassium buffering is often found in astrocytes that surround seizure foci [67]. During seizure-like activity, the concentration of potassium ions rapidly increases around the seizure foci and may not be buffered locally. Residual potassium ions are then taken up by astrocytes and travel a long distance via networks between glial cells. Potassium might be released at locations with lower potassium concentrations and modulate local neuronal activity [68]. Glial networks are formed by gap junction coupling. Calcium waves travel through the glial syncitium, cause glutamate release from astrocytes, and contribute to paroxysmal depolarization shifts [65], [69]. Therefore, blocking gap junction transmission between astrocytes may prevent long-distance synchronization. Our results showed that the extracellular potassium concentration was lowered by CBX application might reflect that the distal release of potassium ions from astrocytes were impaired by the gap junction blocker.

Interneurons are important in synchronizing [14]–[16] and restraining the propagation of seizure-like activity [70], [71]. The prevalence of gap junctions in cortical interneurons suggests that gap junctions play important roles in seizure propagation. We examined 2D-CSD patterns of seizure-like activity after application of the gap junction blocker CBX. The sink signal shifted to layer II/III after the application of CBX, whereas the source signal shifted to layer V. Previous studies found intracortical connections between interneurons in the ACC in layers I and II/III [72]. These results indicate that CBX exerts its effect by disrupting the synchronization of interneurons in the superficial layer. The application of CBX decreased the inhibitory influence in layer II/III, and thus more sink signals appeared.

Gap junctions are involved in oscillations with different frequencies, including theta oscillations [43], [44], gamma oscillations [73], [74], and fast ripples [75]. Previous studies also showed that gap junction blockers blocked carbachol-induced theta oscillations in a brain slice preparation [44], whereas TriMA increased the amplitude of theta oscillations [76]. This was caused by the local synchronization and desynchronization of interneurons. We found that oscillations in the theta frequency range decreased significantly after CBX application, indicating that the activity of local interneurons was desynchronized.

Thirty percent of seizure patients suffer from drug-resistant seizures [77]. One clinical method to cure these patients is DBS. Deep brain stimulation was adapted because it could cure patients with unidentifiable seizure initiation sites, or it could be used to treat patients with seizure foci that could not be removed. One of the targeted brain regions for DBS is the thalamus. The thalamus relays information from the periphery to the central nervous system and is responsible for the synchronization of different cortices. Therefore, some nuclei in the thalamus, such as centromedial, mediodorsal, and parafasicular nuclei, are potential clinical targets for DBS [24], [78]. Previous clinical studies also showed that anterior thalamus stimulation (4–5 V, 90–110 Hz, 60–90 µV) alleviated intractable cingulate seizures [79]. The possible underlying mechanism may be that DBS in the thalamus changes cortical synaptic plasticity [80], [81]. One important feature of thalamocortical afferents is that they contact both excitatory projection neurons and local inhibitory interneurons in the cortex. Thus, somatosensory information is immediately distributed to both excitatory and inhibitory cells. Surprisingly, however, thalamocortical synapses on inhibitory interneurons are much stronger than those on excitatory principal cells [82]. By contacting both inhibitory and excitatory cells, thalamocortical afferents lay the foundation for a simple disynaptic circuit that provides feedforward inhibition. Previous studies also showed that feedfoward inhibition was mediated by EPSCs in the network of gap junction-coupled interneurons [83]. We found that removing the thalamic inputs could potentiate cingulate seizure-like activity [28], indicating that thalamic inputs exert their effect through cortical interneurons. However, under the suppression of CBX, removing the thalamic inputs only partially enhanced seizure-like activity in the ACC. This may indicate that CBX disrupts the synchronicity of interneurons and alleviates the modulatory effect of thalamic inputs on seizure-like activity.

Thalamic inputs terminate at layer II, III and V [84]–[86]. Our previous studies showed that thalamic stimulation induced sink currents in layer V and accompanied source currents appeared in more superficial layer [29], [30]. The sinks current in layer II/III appeared later than that of the initial current in layer V. This CSD pattern indicates that MT afferents terminate on vertically orientated basal dendrites of layer V pyramidal neurons, creating local sink currents and a distal source current at their apical dendrites. Our previous studies showed that the first component of thalamus-evoked activity appeared in layer V near the cingulum [30], suggesting that layer V first receives thalamic inputs. The present study showed that the 2D-CSD profile of the sink signals under the influence of CBX decreased in superficial layers and increased in deep layers, suggesting that inhibitory modulation from the thalamus is weakened in this layer after the application of CBX.
